# Evolutionary insights in Amazonian turtles (Testudines, Podocnemididae): co-location of 5S rDNA and U2 snRNA and wide distribution of Tc1/Mariner

**DOI:** 10.1242/bio.049817

**Published:** 2020-04-28

**Authors:** Manoella Gemaque Cavalcante, Cleusa Yoshiko Nagamachi, Julio Cesar Pieczarka, Renata Coelho Rodrigues Noronha

**Affiliations:** Centro de Estudos Avançados da Biodiversidade, Cytogenetics Laboratory, Institute of Biological Sciences, Federal University of Pará, Belém, Pará, Brazil

**Keywords:** Molecular cytogenetics, Mobile DNA, Karyotype evolution

## Abstract

Eukaryotic genomes exhibit substantial accumulation of repetitive DNA sequences. These sequences can participate in chromosomal reorganization events and undergo molecular cooption to interfere with the function and evolution of genomes. In turtles, repetitive DNA sequences appear to be accumulated at probable break points and may participate in events such as non-homologous recombination and chromosomal rearrangements. In this study, repeated sequences of 5S rDNA, U2 snRNA and Tc1/Mariner transposons were amplified from the genomes of the turtles, *Podocnemis expansa* and *Podocnemis unifilis*, and mapped by fluorescence *in situ* hybridization. Our data confirm the 2n=28 chromosomes for these species (the second lowest 2n in the order Testudines). We observe high conservation of the co-located 5S rDNA and U2 snRNA genes on a small chromosome pair (pair 13), and surmise that this represents the ancestral condition. Our analysis reveals a wide distribution of the Tc1/Mariner transposons and we discuss how the mobility of these transposons can act on karyotypic reorganization events (contributing to the 2n decrease of those species). Our data add new information for the order Testudines and provide important insights into the dynamics and organization of these sequences in the chelonian genomes.

## Abstract (precis)

**Summary:** This paper is important for understanding karyotype evolutionary dynamics in Amazonian turtles, focusing on physical mapping of repetitive DNA sequences.

## INTRODUCTION

The wide variation in the size and organization of eukaryotic genomes is attributed principally to the accumulation of repetitive DNA sequences (Feschotte and Pritham, 2007; Kordis, 2009). Studies suggest that sites rich in repetitive sequences can be critical points for double-strand breaks, non-homologous recombination and chromosomal reorganization in several organisms (Cazaux et al., 2011; Barros et al., 2017; Cavalcante et al., 2018). Moreover, the high mobility of certain sequences (as transposable elements, TEs) can enable them to interrupt the coding sequences of endogenous genes and modify their expression (Kemp and Longworth, 2015; Yin et al., 2018), or be co-opted for the regulation of host genes and thereby interfere with genome function and evolution (McCullers and Steiniger, 2017; Guichard et al., 2018).

The genes encoding the 5S rRNA have the smallest repeating unit length among ribosomal genes (Salina and Adonina, 2018). Due to its conserved character, the 5S rDNA has been widely used as a marker in molecular cytogenetics for the characterization of various species. Most of the investigated karyotypes have relatively few (often just one) 5S rDNA loci (Sochorová et al., 2018; Frade et al., 2019). The 5S rDNA has been reported to co-localize with other multigenes, such as histones genes and small nuclear RNAs (snRNAs) of the U family (Novotná et al., 2011; Piscor et al., 2018). These associations seem to indicate an old and linked organization of such sequences in the genomes of the relevant species (Cabral-de-Mello et al., 2011).

Another group of multigenes often used for mapping in molecular cytogenetics are the snRNAs, which are U2 spliceosomal RNAs. Although the U2 snRNA genes show some sequence conservation, *in situ* mapping reveals that their distribution patterns can be widely diverse among the karyotypes of some groups. For example, the U2 snRNAs can be (i) organized into a single or small number of chromosomal clusters, as reported in fish (Araya-Jaime et al., 2017; Piscor et al., 2018) and some invertebrates (Almeida et al., 2017; Anjos et al., 2018); (ii) arranged in multiple clusters, as observed in some fish (Xu et al., 2017); (iii) dispersed in small copies throughout the genome, as in fish of the family Batrachoididae (Úbeda-Manzanaro et al., 2010); (iv) allocated on supernumerary chromosomes, as noted in the grasshopper, *Abracris flavolineata* (Bueno et al., 2013); and (v) in sex chromosomes, as described in grasshoppers of the subfamily Melanoplinae (Palacios-Gimenez et al., 2013). This broad heterogeneity of chromosomal location observed for U2 snRNA genes may be related to the evolutionary history of the snRNA U family, whose members can behave as mobile elements and exhibit very little conserved synteny (Marz et al., 2008).

The largest group of Class II eukaryotic transposons is composed of members related to the Mariner and Tc1 families (Benjamin et al., 2007). In terms of an organizational pattern, Tc1/Mariner is described as being predominantly dispersed along the karyotypes of several species (Schemberger et al., 2016); however, accumulations have been reported in heterochromatic regions (Ayres-Alves et al., 2017), terminal regions (Schemberger et al., 2016; Gouveia et al., 2017) and sex chromosomes (possibly caused by lack of recombination) (Schemberger et al., 2016). In addition, co-location of rDNA sites with Tc1/Mariner clusters has been observed and it has been proposed that the transposon can participate in rDNA dispersion through recombination events and/or transposition-derived mobilization (Ayres-Alves et al., 2017; Gouveia et al., 2017).

The order Testudines is considered one of the oldest lineages among existing vertebrates (Ferri, 2002). Cytogenetic studies have revealed wide karyotypic variation among their representatives (2n=26–68), which is attributed mainly to the number of microchromosomes (Montiel et al., 2016; Cavalcante et al., 2018). Species of genus *Podocnemis* (Pleurodira, Podocnemididae) present the second smallest diploid number in the order (2n=28) (Noronha et al., 2016; Cavalcante et al., 2018). Cytogenomic studies indicate a derived condition for *Podocnemis* and suggest that multiple fusions involving microchromosomes may have been responsible for the reduction of 2n in this genus (Montiel et al., 2016; Cavalcante et al., 2018).

In turtles, cytogenomic studies based on multiple-copy DNA sequences have provided important data on the dynamics of these sequences and how they can interfere with the genomic organization of the group. Although the organizational dynamics of 5S rDNA, U2 snRNA and Tc1/Mariner have been studied in different groups of animals, such data are limited among reptiles (Sochorová et al., 2018). At present, no *in situ* mapping data are available for these sequences in turtles (order Testudines). Here, we report the chromosomal locations of the 5S rDNA, U2 snRNA, and Tc1/Mariner sequences in the turtle species, *Podocnemis expansa* and *Podocnemis unifilis*, and seek to further understand the genomic organizations among reptiles and identify evolutionary factors that may be involved in the wide karyotypic diversity of the order Testudines.

## RESULTS

Both species presented a diploid number of 28 chromosomes. *Podocnemis expansa* had a fundamental number (FN) of 54 and a karyotype formula of 24 m/sm+2st+2a, while *P. unifilis* presented with FN=52 and a karyotype formula of 22 m/sm+2st+4a.

**Fig. 1.**
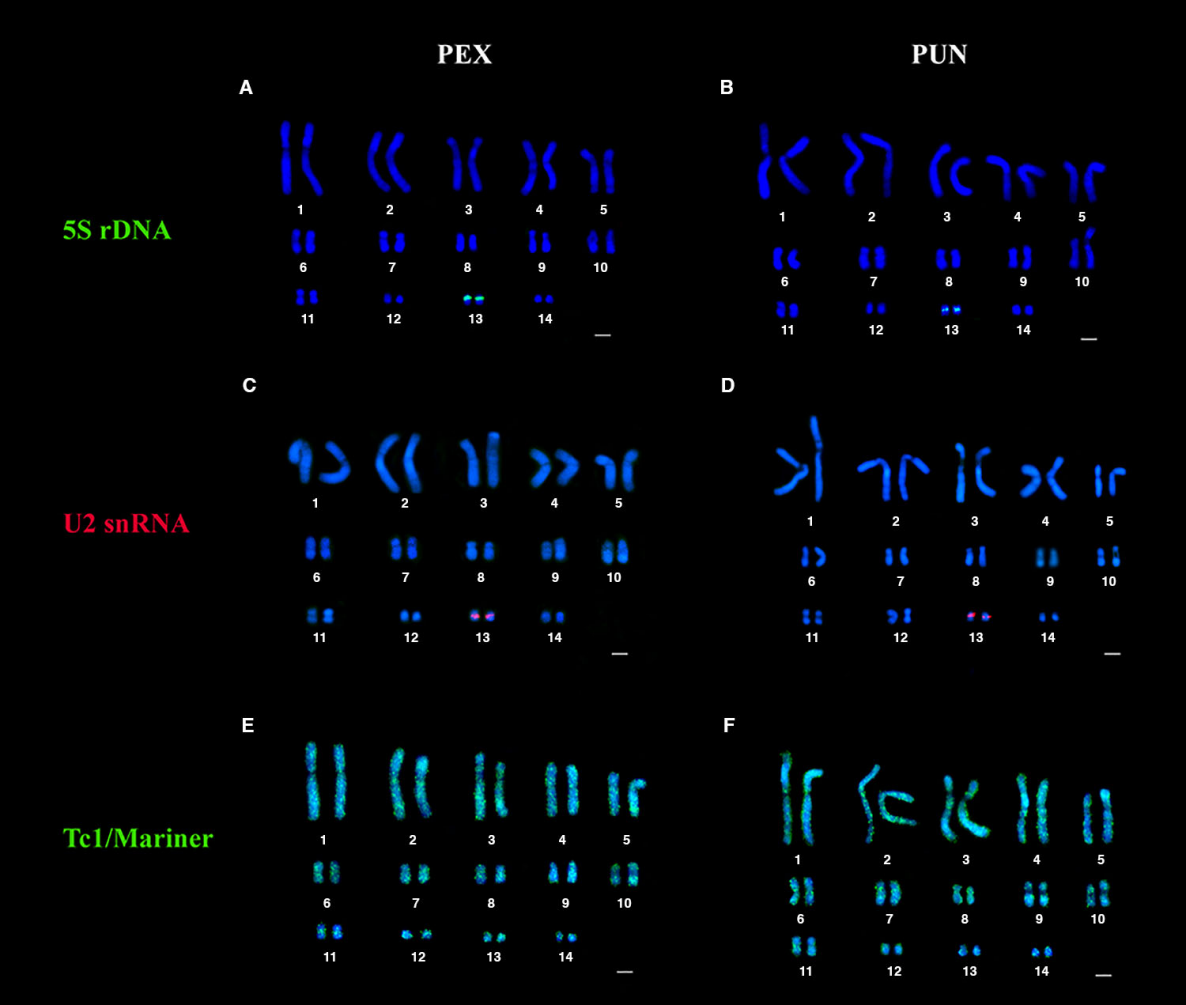
**Physical mapping of repetitive DNA.** The codes PEX and PUN refer to the karyotypes of *P. expansa* and *P. unifilis*, respectively. The 5S rDNA (green) is detected in a single chromosomal pair (pair 13) in (A) PEX and (B) PUN. Mapping of the U2 snRNA (red) also yields signals on chromosome pair 13 for (C) PEX and (D) PUN. The transposon, Tc1/Mariner (green), is widely distributed along the karyotypes of (E) PEX and (F) PUN.

*In situ* mapping with 5S rDNA probes revealed pericentric signals in a single chromosomal pair (pair 13) for both *P. expansa* and *P. unifilis* (Fig. 1A,B)*.*

Hybridizations of U2 snRNA were observed in the pericentric region of the same chromosomal pair as the 5S rDNA (pair 13) for both species (Fig. 1C,D).

**Fig. 2.**
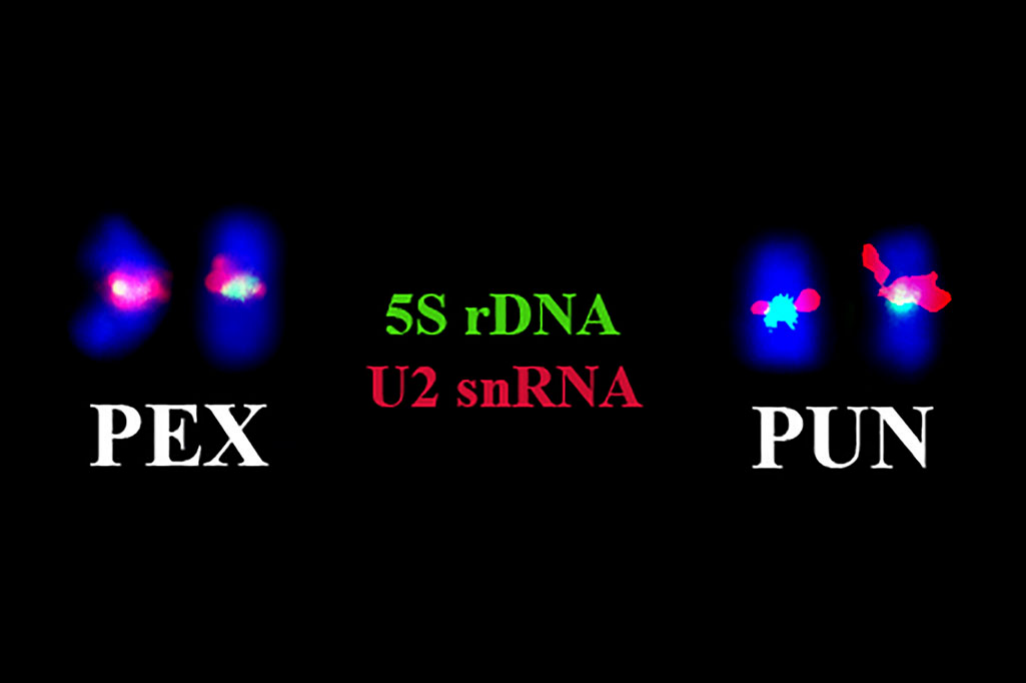
**Double-FISH with 5S rDNA and U2 snRNA probes.** Double-FISH using probes for the 5S rDNA (green) and U2 snRNA (red) reveals that these sequences co-localize similarly in chromosome pair 13 of *P. expansa* (PEX) and *P. unifilis* (PUN).

Double-FISH using 5S and U2 probes revealed that these signals co-localized similarly in chromosome pair 13 of both species (Fig. 2).

The transposon, Tc1/Mariner, was widely distributed along the karyotypes of *P. expansa* and *P. unifilis.* The Tc1/Mariner signal was predominantly dispersed throughout the euchromatic region, with pericentric accumulations on some chromosomal pairs. Heterochromatic regions of chromosome pair 10 of *P. expansa* and pairs 9 and 10 of *P. unifilis* had low signal intensities for Tc1/Mariner (Fig. 1E,F).

## DISCUSSION

The genus *Podocnemis* has the second-smallest diploid number among the chelonians. Our results corroborate previous reports that *P. expansa* and *P. unifilis* (Montiel et al., 2016; Noronha et al., 2016) have a diploid number of 2n=28 chromosomes. Molecular cytogenetics studies indicate that the smaller diploid numbers of *Podocnemis* represent a derived condition; multiple fusions involving microchromosomes appear to be responsible for the reduction of the diploid number (Montiel et al., 2016; Cavalcante et al., 2018). Previously, Cavalcante et al. (2018) demonstrated evidence of possible chromosomal fusions in these species. Multiple interstitial telomeric signals are seen on seven chromosomal pairs of *Podocnemis* (pairs 1­­–5, 7 and 13), suggesting chromosomal sites that may be involved in these genomic reorganization events. Mobile elements are often found at chromosomal break points (Barros et al., 2017). Therefore, it is likely that TEs such as Tc1/Mariner, which is widely distributed throughout the genomes of *P. expansa* and *P. unifilis*, were involved in the karyotypic reorganizations thought to have occurred in these species*.*

Although only limited *in situ* rDNA location data are available for other reptiles, the 18S and 5S rDNAs are typically found in only one chromosomal pair: 18S sequences are usually found in a microchromosome, while the 5S sequences may be found in a macro- or microchromosome (Srikulnath et al., 2015). Cavalcante et al. (2018) reported that the 45S rDNA sequences of *P. expansa* and *P. unifilis* are located in the pericentric region of the first pair of macrochromosomes, in association with interstitial telomeric sequence regions. The authors proposed that the 45S rDNA was located on a pair of microchromosomes early during the karyotypic evolution of these species (similar to the location described for more basal chelonian species, which have high 2n), and it was subsequently relocated to the first pair of macrochromosomes through fusion events. The present report offers the first information about the 5S rDNA locus in turtles. Our results demonstrate the conserved character of a single gene locus (chromosome pair 13) in *P. expansa* and *P. unifilis* and indicate that despite the genomic reorganization proposed for *P. expansa* and *P. unifilis*, there is a high conservation of 5S genes in smaller chromosomes across *Podocnemis*. In other reptiles (as in squamates), the 5S rDNA is also present in only one chromosome pair, although with different chromosomal locations (Srikulnath et al., 2011; 2015). In fish, Martins and Galetti-Junior (1999) proposed that the presence of 5S rDNA in only one pair of chromosomes represents the ancestral condition. This seems also to be the case for reptiles. We further suggest that a strong purifying selection acts on 5S rDNA clusters, preventing these multigenes from spreading in the genomes of *P. expansa* and *P. unifilis*.

The snRNA U2 signals were observed in the same chromosomal pair as the 5S rDNA in *P. expansa* and *P. unifilis* (chromosome pair 13)*.* Cavalcante et al. (2018) previously reported that chromosome pair 13 presented pericentromeric signs of histone H3 genes in *P. expansa* and interstitial telomeric sequences in *P. expansa* and *P. unifilis*. Thus, chromosomal pair 13 seems to harbor multiple repetitive sequences. In animal genomes, the association/co-location of multigene families has been reported for rRNAs, histones genes and snRNAs (Cabral-de-Mello et al., 2011; Cavalcante et al., 2018). According to studies by Dover (1989) and Liu and Fredga (1999), these links between multigenes are important for maintaining the conservation of multiple matrices. It has also been hypothesized that these associations among multigene families may play a functional role in nuclear organization (Kaplan et al., 1993; Cabral-de-Mello et al., 2011).

The wide dispersion observed for Tc1/Mariner in *P. expansa* and *P. unifilis* is consistent with that previously described for transposon (Schemberger et al., 2016). Heterochromatic accumulations of Tc1/Mariner have also previously been reported in some fish (Ayres-Alves et al., 2017; Gouveia et al., 2017). This may indicate that there is a selection pressure against inserting TEs into euchromatin; this could reflect ectopic exchanges (Oliveira et al., 2013) and the low recombination rates of these regions, which reduces insertion damage (Delaurière et al., 2009). It is expected that transposable elements active and recently acquired will be preferentially located in the euchromatin (Oliveira et al., 2013). Therefore, the Tc1/Mariner sequences present in *P. expansa* and *P. unifilis* can be considered recent, due to their wide euchromatic dispersion and few heterochromatic accumulations. It is important to highlight the existence of considerable intragenomic heterogeneity among the TEs; the amplification products of TEs are mixtures of various genomic sequences of unknown composition. Therefore, the derived probes would hybridize to different genomic locations at different intensities. If, as is possible, this occurred in the present study for Tc1/Mariner hybridizations, it would explain why some signals were weak while others were strong, and some signals were clustered while others were dispersed.

Noronha et al. (2016) demonstrated wide distribution of the retrotransposon, Rex 6, in euchromatin of *P. expansa* and *P. unifilis*. The authors emphasized that TE mobility can produce structural changes, trigger chromosomal rearrangements and modify gene regulation patterns. In general, TEs are present as non-autonomous copies in the genomes that are generated by a degradation process (Fernández-Medina et al., 2012). However, it is possible to detect transcriptional activity at the limits of degenerated sequences, as demonstrated in the fish family, Parodontidae (Schemberger et al., 2016), where molecular cooptation of these sequences was detected even after their inactivation. In this sense, given the intense euchromatic presence of Tc1/Mariner in *P. expansa* and *P. unifilis*, it is possible to infer that transposon activity may alter gene regulation, confer new genomic functions and/or act on the karyotypic reorganization events that resulted in the decrease of 2n in these species.

### Conclusion

We herein demonstrate that the gene locus number for the 5S rDNA is highly conserved (only one chromosome pair) in *P. expansa* and *P. unifilis*. A similar result was previously obtained for the 45S rDNA loci of these species (although in distinct chromosome pairs), suggesting that a low number of rDNA loci is consistent in this group and represents a plesiomorphic character. We also demonstrate that there are links between the multigenes, 5S rDNA and U2 snRNA, which likely act to maintain their matrices in these species. Finally, we show that Tc1/Mariner is widely dispersed along the karyotypes of the species (preferentially in euchromatic regions). Based on this, we suggest that these transposons may alter gene regulation, have their degenerate sequences co-opted for new genomic functions and/or participate in karyotypic reorganization events. Such data have not previously been reported for the order Testudines, and our findings provide important insight into the dynamics and organization of these repetitive sequences in chelonian genomes.

## MATERIALS AND METHODS

### Specimens and ethics committee approval

The biological materials of *P. expansa* and *P. unifilis* specimens were collected at the Zoobotanical Park, Mangal das Garças, Belém, Pará, Brazil. Five males and five females were analyzed for each species. This study was conducted in accordance with ethical recommendations for the use and management of turtles in research, under a protocol approved by the Ethics Committee on Experimental Animal Research (license number 68–2015) and the Biodiversity Information and Authorization System (SISBIO; license number 42642–5).

### Chromosome preparation and probe production

Lymphocyte culture and chromosomal preparations were performed as described by Viana et al. (2016). Genomic DNA was extracted using GenElute™ Mammalian Genomic DNA Miniprep Kit (Sigma-Aldrich, St Louis, MO, USA). The genes encoding 5S rDNA, U2 snRNA and Tc1/Mariner were amplified by polymerase chain reaction (PCR), using the following primers: 5S rDNA, 5rF (5′-GCC ACA CCA CCC TGA ACA C-3′) and 5rR (5′-GCC TAC GAC ACC TGG TAT TC-3′) (Suárez et al., 2017); U2 snRNA, 5′-TCT CGG CCT (AT) (AT)T GGC TAA-3′ and 5′-G(AC)G GTA (GC) TG CAA TAC CGG-3′ (Colgan et al., 1998); and Tc1/Mariner, MAR-188F5′-ATCTGRAGCTATAAATCACT and MAR-251R 5′-CAAAGATGTCCTTGGGTGTG (Lampe et al., 2003).

The reaction mixtures contained 80 ng genomic DNA, 0.2 μM of each primer, 0.16 mM dNTPs, 1 U Taq DNA Polymerase (Invitrogen), 1.5 mM MgCl_2_ and reaction buffer 10x (200 mM Tris, pH 8.4, 500 mM KCL). The amplification conditions were as follows: 4 min - 95°C/(1 min - 95°C/1 min - 60°C/2 min - 74°C) for 35 cycles/5 min - 74°C for 5S rDNA and Tc1/Mariner; and 4 min - 95°C/(1 min - 95°C/1 min - 57°C/2 min - 74°C) 30 cycles/5 min - 74°C for U2 snRNA*.* The amplifications generated bands with the following sizes: 120 bp for 5S rDNA; 220 bp for U2 snRNA; and multiple bands (300, 500 and 1000 bp) for Tc1/Mariner.

The probes were labeled by nick-translation with biotin 14-dATP or digoxigenin 16-dUPT using a BioNick Labeling System (Invitrogen) and a DIG-Nick kit (Roche Applied Science), respectively.

### Fluorescence *in situ* hybridization (FISH)

FISH was performed as described by Pinkel et al. (1986), with some adaptations. Signals were detected with avidin-CY3 (Sigma-Aldrich) and antidigoxigenin-FITC (Roche). Chromosomes were counterstained with 4′,6-diamidino-2-phenylindole (DAPI; 0.2 μg ml^−1^) in Vectashield H-100 mounting medium (Vector) and analyzed under an epifluorescence microscope (Nikon H550S).
